# Transfer Function Models for the Localization of Seizure Onset Zone From Cortico-Cortical Evoked Potentials

**DOI:** 10.3389/fneur.2020.579961

**Published:** 2020-12-10

**Authors:** Golnoosh Kamali, Rachel June Smith, Mark Hays, Christopher Coogan, Nathan E. Crone, Joon Y. Kang, Sridevi V. Sarma

**Affiliations:** ^1^Neuromedical Control Systems Laboratory, Department of Electrical and Computer Engineering, Institute of Computational Medicine, Johns Hopkins University, Baltimore, MD, United States; ^2^Neuromedical Control Systems Laboratory, Department of Biomedical Engineering, Institute of Computational Medicine, Johns Hopkins University, Baltimore, MD, United States; ^3^Cognitive Research, Online Neuroengineering and Electrophysiology Laboratory, Department of Biomedical Engineering, Johns Hopkins University, Baltimore, MD, United States; ^4^Cognitive Research, Online Neuroengineering and Electrophysiology Laboratory, Department of Neurology-Epilepsy, Johns Hopkins School of Medicine, Baltimore, MD, United States; ^5^Department of Neurology-Epilepsy, Johns Hopkins School of Medicine, Baltimore, MD, United States

**Keywords:** epilepsy, CCEPs, stimulation, SPES, seizure

## Abstract

Surgical resection of the seizure onset zone (SOZ) could potentially lead to seizure-freedom in medically refractory epilepsy patients. However, localizing the SOZ can be a time consuming and tedious process involving visual inspection of intracranial electroencephalographic (iEEG) recordings captured during passive patient monitoring. Cortical stimulation is currently performed on patients undergoing invasive EEG monitoring for the main purpose of mapping functional brain networks such as language and motor networks. We hypothesized that evoked responses from single pulse electrical stimulation (SPES) can also be used to localize the SOZ as they may express the natural frequencies and connectivity of the iEEG network. To test our hypothesis, we constructed patient specific transfer function models from the evoked responses recorded from 22 epilepsy patients that underwent SPES evaluation and iEEG monitoring. We then computed the frequency and connectivity dependent “peak gain” of the system as measured by the $H_{\infty }\;$norm from systems theory. We found that in cases for which clinicians had high confidence in localizing the SOZ, the highest peak gain transfer functions with the smallest “floor gain” (gain at which the dipped $H_{\infty }\;$ 3dB below DC gain) corresponded to when the clinically annotated SOZ and early spread regions were stimulated. In more complex cases, there was a large spread of the peak-to-floor (PF) ratios when the clinically annotated SOZ was stimulated. Interestingly for patients who had successful surgeries, our ratio of gains, agreed with clinical localization, no matter the complexity of the case. For patients with failed surgeries, the PF ratio did not match clinical annotations. Our findings suggest that transfer function gains and their corresponding frequency responses computed from SPES evoked responses may improve SOZ localization and thus surgical outcomes.

## Introduction

Epilepsy is a widespread neurological disease that affects nearly 1% of the world's population (1). First-line treatment for epilepsy is anti-epileptic medication, however up to 30% of patients do not respond to the drugs and thus are considered to have medically refractory epilepsy (MRE) (2, 3). However, for MRE patients with well-defined seizure onset zones (SOZ) and early spread regions, seizure freedom may be possible with surgical resection, disconnection of, or electrical stimulation of the SOZ. Localization of the SOZ often requires invasive monitoring with intracranial (iEEG) recordings, but even with such techniques surgical success rates remain highly variable ranging 30–70% (4, 5).

Current SOZ localization methods rely on clinicians inspecting abnormalities on individual channels from iEEG recordings, despite the fact that the epileptic brain is a complex network wherein individual channels interact dynamically (6); therefore, novel tools that investigate epilepsy with a network model may serve as a superior framework for identifying the SOZ. A clinical tool developed to analyze brain networks *in vivo* is single-pulse electrical stimulation (SPES), which elicits an evoked response potential (ERP) in regions that are connected to the stimulation site, known as cortico-cortical evoked potentials (CCEPs) (7, 8).

SPES evokes CCEPs (9, 10) to define effective, or directed, connections to map the human brain (10). The mechanisms of CCEPs induced by SPES are still not fully certain (10), but it is hypothesized that the earliest sensory response is a depolarization in the middle laminae (N1 response), followed by complex patterns of excitatory and inhibitory post-synaptic potentials to form the N2 response (11). The technique was first used to map inter-areal connectivity of the language (9) and motor cortices (12), but has been extended to evaluate functional connections of the frontal-temporal lobe (13), the parietal-frontal lobe (14), the limbic network (15), the insula (16, 17), and deeper brain structures (18, 19). In the last decade SPES has been gaining traction as a tool to probe functional and pathological connectivity in epilepsy and to localize the epileptic networks (20).

SPES has been used as an investigational tool in epilepsy by probing seizure networks as well as investigating cortical excitability (20). A decreased threshold of excitability, as measured by the presence or strength of CCEPs in the stimulating or surrounding regions, possibly indicates seizure-prone tissue (20). This increased excitability is hypothesized to be evident when features of CCEPs differ when stimulated or recorded in the SOZ regions as compared to in healthy tissue. For example, the amplitude of the CCEP response was found to be higher in the SOZ regions when compared to outside regions (21–23) as well as in early ictal propagation sites (24, 25). A second marker of epileptogenicity induced by CCEPs are “delayed responses,” neuronal activities that resemble spikes or slow waves that occur 100 ms to 1 s after the stimulation onset that are more likely to be present in SOZ regions (26–28). Additionally, it was shown that removal of areas that consistently exhibited these delayed responses resulted in good outcomes (29–31). High frequency activity during the CCEP (32, 33) or suppression of high-frequency activity after stimulation (34) have been investigated for their SOZ localizing power. Specifically high frequency oscillations were found to colocalize with CCEP responses (35–37), in one study as much as 40% of the time (38). Lastly, graph theoretical properties of the networks generated from CCEP response amplitudes revealed that networks are more bi-directionally connected in the SOZ than in non-SOZ regions (39–41).

Current computational approaches to analyzing seizure networks from CCEPs either compute iEEG features on individual channels, such as the N1 peak amplitudes and signal latencies (21, 25, 36, 42, 43), or they compute static pairwise correlations, organize these correlations into adjacency matrices, and derive graph-theoretic measures (44, 45). These approaches are limited in their ability to capture the underlying network dynamics of the disease. Computing iEEG features such as N1 peak amplitudes forgoes the network aspect of epilepsy by inspecting individual channels instead of its connections to others. While graph theoretic approaches can compute summary statistics of interest such as nodal centralities and network hubs, such measures are not based on well-formulated hypotheses of the role of the SOZ in the iEEG network, and worse many different networks (adjacency matrices) can have identical summary statistics. The interpretations of such measures are thus ambiguous. In contrast, dynamical network models can reveal the natural frequencies of the epileptic network, its connectivity properties, and the underlying dynamics of seizure generation.

We hypothesized that the SOZ is distinguishable from other brain regions in that it generates the “largest” network response to the “smallest” pulse input or “kick.” To test this hypothesis, we investigated a property of transfer functions that reflected the epileptogenic nature of the EEG network. Specifically, we calculated the system gain defined by the $H_{\infty }$ norm of a transfer function, a notion that describes the amplification and spread of the CCEPs in the network, and the corresponding input “size” required to achieve the system gain. Our approach consisted of building patient specific transfer function models for every stimulation pair. System gains were then computed by calculating the $H_{\infty }$ norm of the single input-multi output (SIMO) model, the “peak,” for each stimulation pair. For each system model, the 2-norm or “energy” of the associated frequency response a the roll-off, defined to be 3dB below DC gain, was also computed and denoted as the “floor” gain. Finally, we calculated the peak-to-floor (PF) gain ratio. We then defined a confidence statistic that was computed for each patient to assess the level of agreement between the PF ratio, the stimulated SOZ contacts, and surgical outcome. We found that the PF ratio correlates well with clinically annotated SOZ and early spread regions for more straightforward clinical cases and with greater accuracy than current visual assessment approaches. This computational tool may aid clinicians in the identification of the epileptogenic network and thereby improve surgical outcomes.

## Materials and Methods

### Patients

We used a retrospective dataset of 22 MRE patients who underwent iEEG monitoring and SPES for localization of seizures at the Johns Hopkins Hospital (JHH) Epilepsy Monitoring Unit (EMU) with patient's consent as part of the Studies of Patients with Implanted Intracranial Electrodes (IRB 00044461). At least two board-certified epileptologists reviewed the iEEG during the patient's seizures and identified electrodes involved in regions of seizure onset (SOZ), early spread (EP), and irritative (IZ). Seizure onset was defined as the first consistent presence of rhythmic spikes, rhythmic sharp waves, regular or low amplitude activity in the beta range, or recruiting gamma activity that was either prior or coinciding with the clinical manifestation of the seizure. The early spread regions were defined as those areas to which the seizure activity spread before secondary generalization occurred, and irritative zones were marked where there were epileptic spikes only (46).

**Figure 1:**
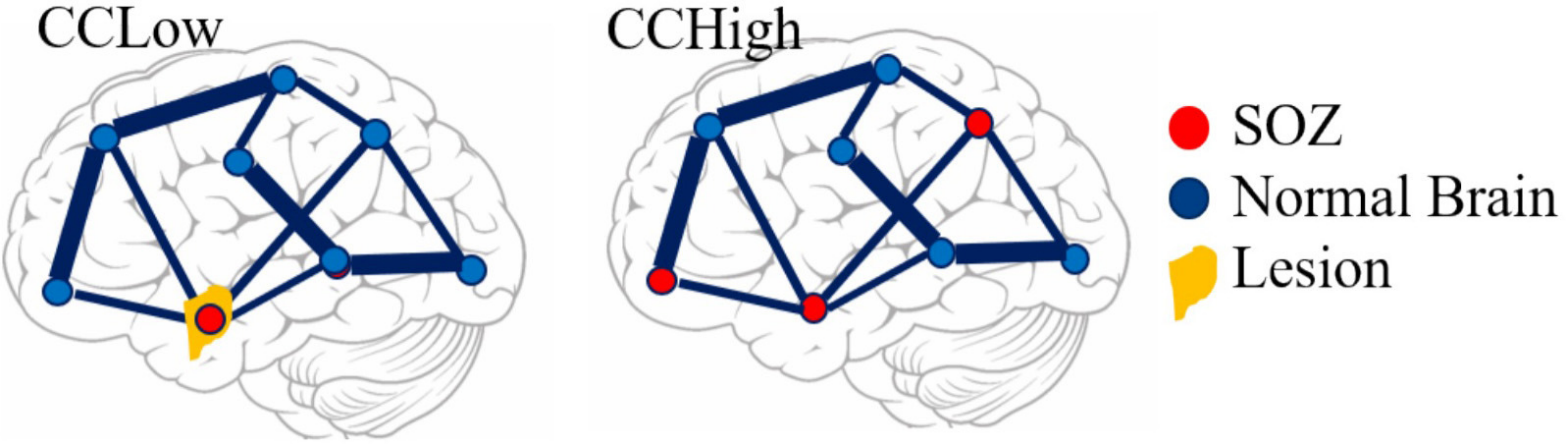
Pictorial representation of clinical complexity. CCLow was defined as cases that were lesional, only one seizure focus, or solely confined to the temporal lobe. CCHigh was defined as cases of multi-focal epilepsy, a seizure focus outside the temporal lobe, and/or no lesions on imaging.

Patients were classified as having successful surgical outcomes if they experienced seizure freedom one-year after surgery (Engel class I) or nearly seizure freedom (Engel class II) and failed outcomes if they experienced seizure recurrence (Engel classes III-IV) (47) (Table 1). However, in instances where a responsive neurostimulation device (RNS) was used rather than resection or ablation, Engel class III was a surgical success. Thirteen of the 22 patients underwent surgical intervention and were evaluated for surgical outcome. Due to the lack of outcome data in the remaining 9 patients, we categorized all patients by a custom “clinical complexity (CC)” score (Figure 1) (42, 43). Patients with lesional or focal epilepsy in the temporal lobe were classified as CCLow and those patients with non-lesional or multifocal epilepsy outside of the temporal or that were non-localizable were classified as CCHigh (Figure 1). These categories were developed in light of previous outcome studies that showed that patients with visible lesions on MRI (lesional) have higher surgical success rates (~70%), while non-lesional, extratemporal, and multifocal epilepsies have much lower success rates (48–50) (Figure 1).

**Table 1:** Summary of patient clinical data.

**Patient**	**Gender**	**Age**	**Seizure type**	**MRI**	**SOZ**	**Surgery**	**Pathology**	**CC**	**ES**	**# of SOZ contacts**	**Total # of stimulated contacts**
1	M	25	FocalA, FocalA to BilateralTC	Non-lesional	Left frontal, involving the premotor and motor cortex	Resection	Non-specific, inflammatory changes	High	3	3	7
2	F	43	Focal_IA	Non-lesional	Left posterior basal temporal-occipital region	Resection	Normal	High	3	3	8
3	F	35	Focal-IAsensory Focal_IA	Cystic multilobulated cortically based mass in left temporal lobe	Left temporal	Resection	DNET vs. oliogodendroglioma	High	2	1	5
4	F	18	FocalA to BilateralTC	Subtle thickening in the right middle frontal gyrus	Right frontal	Resection	Cortical dysplasia	Low	1	3	11
5	M	32	Focal-Asensory Focal-Amotor Focal_IA	Gliososis in the posterior superior left parietal lobe	Left superior parietal lobule	Resection	Cortical dysplasia	High	4	8	25
6	M	38	Focal_IA with occasional TC	Mild asymmetric thickening in the dorsal left para-hippocampal gyrus	Left mesial and para-hippocampal gyrus	MRgLiTT	N/A	Low	1	4	5
7	F	32	Focal_IA	Left frontal encephalomalacia	Left posterior cingulate	MRgLiTT	N/A	High	4	3	26
8	F	27	Focal_IA	Bilateral occipital lissencephaly	Bilateral mesial temporal structures	RNS	N/A	High	3	8	13
9	F	24	Focal_IA	Left temporal encephalomalacia	Left temporal lobe	Resection	Non-specific, inflammatory changes	Low	1	2	9
10	F	27	Focal_IA to BilateralTC	Left periventricular hetertopia and left frontal encephalamalacia	Left inferior frontal region	Resection	Non-specific, inflammatory changes	High	1	4	19
11	F	51	Focal_IA BilateralTC	Right MTS	Right mesial temporal structures	MRgLiTT	N/A	Low	N/A	2	6
12	M	48	Focal_IA	Periventricular bilateral nodular heterotopa and diffuse cortical dysgensis	Left mesial temporal structures	MRgLiTT	N/A	High	2	2	17
13	F	23	Focal_IA	Left temporal encephalomalacia	Left temporal lobe, mesial and neocortical	RNS	N/A	Low	3	5	18
14A	F	23	Focal_IA	Non-lesional	Right posterior temporal region	Awaiting surgery	N/A	High	N/A	3	25
14B	F	23	Focal_IA	Non-lesional	Right posterior temporal region	Awaiting surgery	N/A	High	N/A	3	10
15	M	32	Focal_IA with and without BilateralTC	Non-lesional	Right temporal lobe (neocortex)	Awaiting surgery	N/A	High	N/A	1	9
16	M	32	Focal_IA	Right parietal encephalomalacia	Right temporal and parietal region	Awaiting surgery	N/A	High	N/A	5	14
17	M	19	Focal_IA with occasional TC	Non-lesional	Left mesial temporal structures	Awaiting surgery	N/A	Low	N/A	4	17
18	F	35	Focal_IA	Right MTS	Bilateral mesial temporal structures	MRgLiTT	N/A	Low	1	4	19
19	M	58	Focal_IA	Non-lesional	Left mesial temporal structures	Awaiting surgery	N/A	High	N/A	2	17
20	M	41	Focal_IA	Enlargement of the left amygdala	Left temporal neocortex	Awaiting surgery	N/A	High	N/A	5	18
21	F	32	Focal_A Focal_IA	Left occipital periventricular nodules	Left occipital and right posterior temporal region	Awaiting surgery: Possible RNS	N/A	High	N/A	2	15
22	F	53	Focal_IA with secondary generalization	Non-lesional	Left temporal lobe (temporal pole and mesial temporal structures	Awaiting surgery	N/A	Low	N/A	7	25

*FocalA, Focal Awareness Seizure; Focal_IA, Focal Impaired Awareness Seizures; TC, Tonic Clonic Seizures; MRgLiTT, MRI guided laser interstitial thermal therapy; RNS, Responsive Neurostimulator*.

### Single-Pulse Electrical Stimulation

SPES was conducted in a bipolar fashion of adjacent electrode pairs clinically annotated in the SOZ and early spread regions as well some outside of the SOZ using a Blackrock acquisition system at a sampling rate of 1 or 2 kHz (51). A monophasic, alternating polarity, 0.3 ms width square wave pulse at a fixed frequency of 0.5 Hz was delivered to all the electrode pairs an average of 50 times. Current intensity was titrated until manifestations of local/distant evoked response potentials (ERPs), discharges/seizures, or a maximum intensity of 12 mA was reached. A 5 mA stimulus intensity was most often used. Responses were recorded from all channels during the 50 trials. The data was digitized and stored in an IRB-approved database compliant with the Health Insurance Portability and Accountability Act regulations. Data was then preprocessed as .dat files for analysis in MATLAB (52). The research protocol was approved by the Institutional Review Board and informed consent was obtained from all participants.

### iEEG Preprocessing

**Figure 2:**
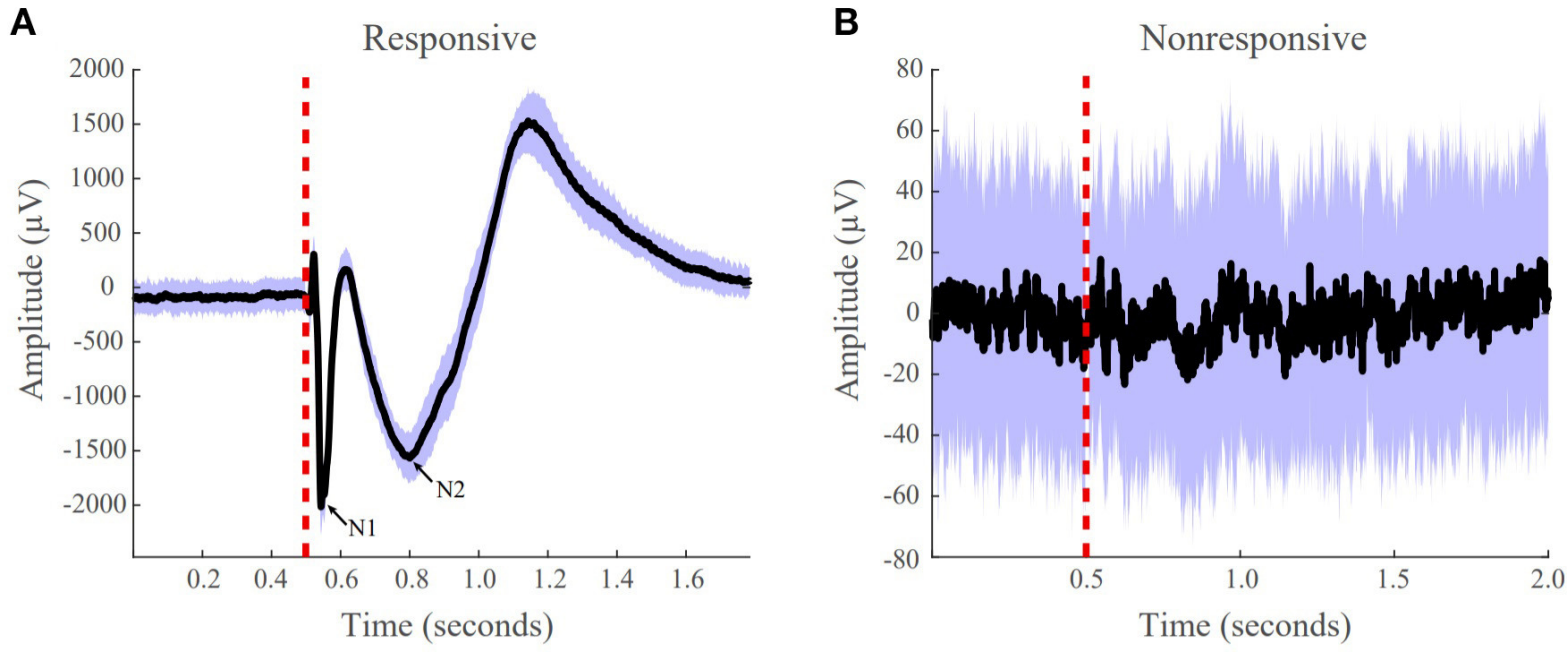
Examples of responsive and non-responsive waveforms from patient iEEG data. **(A)** Responsive CCEPs were defined as an absolute value of the post-stimulus amplitude >100 uV from the baseline. The N1 and N2 peak are labeled. **(B)** Non-responsive CCEP that does not meet the 100 uV post-stimulus amplitude threshold. The black line indicates the average evoked response, the purple boundaries denote one standard deviation, and the red vertical dotted line indicates the stimulation onset.

The average evoked response was computed for every contact in 2 s epochs. We included 500 ms of data before stimulus onset and 1,500 ms post stimulus onset. We calculated the distribution of the 50 time series responses for each 2 s window and marked channels as artifactual if the median standard deviation of the sample distributions was >1,000. These artifactual channels were then removed from the dataset. Next, artifacts due to electrical stimulation were removed by replacing the data 2 ms before and 8 ms after stimulus onset with a linearly spaced vector between those voltage values. We classified channels as responsive and non-responsive if the absolute value of the maximum post-stimulus amplitude was >100μ*V* from the baseline (Figure 2). Non-responsive channels were removed from the dataset before model construction.

### Transfer Function Model Construction

**Figure 3:**
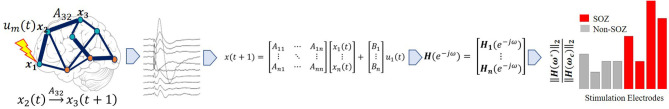
Pipeline to obtain system gain ***H***_∞_- to- input norm ratio from individual patients' SPES iEEG data. Starting from top left, a brain region is stimulated. Then a stereotyped response is extracted for each electrode, from which the average evoked CCEP is calculated. The CCEPs are used to construct a state-space model. From the state space model, a SIMO transfer function vector is constructed via ***H***(*z*) = ***C***(*zI* − ***A***)^−1^***B***. Then the maximum system gain is calculated for every stimulation pair through ‖***H***(ω)‖_∞_ = *sup ωϵRσ*_max_(***H***(*e* − *iω*)) and the 2-norm of the associated frequency response at the cutoff frequency, is computed resulting in the PF ratio. The expectation is that the true SOZ contacts will have the largest PF ratios.

SIMO transfer functions were constructed for each subject and each stimulating electrode pair to estimate the behavior of the CCEPs. To construct our transfer function models, we first built stable, discrete, linear time invariant (LTI) state space models of the following form for each stimulating contact pair:

(1) $$\begin{array}{l} x\left(t+1\right)=Ax\left(t\right)+Bu\left(t\right) \end{array}$$

where ***x***(*t*) ϵ ℝ^*N*×1^ is the state vector, ***A*** ϵ ℝ^*N*×*N*^ is the state transition matrix, *u*(*t*) ∈ ℝ is the input stimulation pulse, and ***B*** ϵ ℝ^*N*×1^ is the input matrix, with *N* representing the total number of contacts for each dataset. ***A*** and ***B*** were calculated via least-squares estimation as described in (53), and the state vector was comprised of the responsive iEEG signals (Figure 3). The models were stimulated with input signal (*t*) = 0 or 1, where the first non-zero element corresponded to the iEEG stimulation onset, with a pulse duration of 2 ms, and *t* is the index for each millisecond. The pair of stimulation electrodes were not included in the models as state variables and were instead characterized as providing the exogenous input *u*(*t*). Next, to improve our model fits, we established a scaling factor, α, based on the range of data **x**(*t*) in relation to the range of the model reconstruction $\hat{\text{x}}\left(t\right)$ for every contact, *k*:

(2) $$\begin{array}{l} \alpha_{k}=\frac{\max\;x_{k}\left(t\right)-\min\;x_{k}\left(t\right)}{\max\;\hat{x_{k}}\left(t\right)-\min\;\hat{x_{k}}\left(t\right)} \end{array}$$

We then scaled our ***A*** and ***B*** by this factor, giving us the following:

(3) $$\begin{array}{l} \bar{x}\left(t+1\right)=\bar{A}x\left(t\right)+\bar{B}u\left(t\right) \end{array}$$

where $\bar{A}=\;\alpha A$ and $\bar{B}=\alpha B$.

Once the state space models were created, we calculated the SIMO transfer function model from *u*(*t*) to ***x***(*t*) via the formula

(4) $$\begin{array}{l} H\left(z\right)={\left(zI-\bar{A}\right)}^{-1}\bar{B} \end{array}$$

which is derived by taking the z-transform of (1). These transfer function models represent the input-output behavior of CCEPs under SPES. The SIMO transfer function models characterize how each iEEG node (channel) *dynamically* influences the rest of the network and how the network responds to an exogenous stimulus, like SPES.

### Investigating Model Properties

After constructing the SIMO transfer function models for each stimulating pair for each subject, we investigated whether properties of these transfer functions correlated to clinically annotated SOZ regions. Specifically, we investigated the peak system gain and the magnitude of the frequency response at the roll-off frequency defined to be the frequency at which the magnitude dipped 3dB below the DC gain (gain at 0 frequency) for each model. The *system gain* of a transfer function, a metric that quantifies how much ERPs can be amplified and spread in the iEEG network, may reveal epileptogenic zone (EZ) regions. The larger the gain, the more influence the node has on spreading activity throughout the network. We also hypothesized that the peak of the frequency response should be followed by a steep roll-off since seizures happen infrequently, which is likely a consequence of resonance in the iEEG network. Consistent with the theory of “fragility” in epileptic brain networks (54), we further hypothesized that the SOZ should produce the largest network responses with the smallest input size.

We therefore proposed a metric which can capture the large system responses and its fast magnitude drop-off through a ratio of peak-to-floor gains, the PF ratio. Epileptogenic regions when stimulated should result in a high PF ratio. To compute the PF ratio for a given stimulation pair, we calculated the system gain of all the SIMO transfer function as quantified by the $H_{\infty }$ norm. The $H_{\infty }$ norm of each stimulation electrode pair *j* was calculated, as follows:

(5) $$\begin{array}{l} {\left\|H_{\text{j}}\right\|}_{\infty }=sup\;\omega \epsilon R\sigma_{\max\;}\left(H_{j}\left(e-i\omega \right)\right)=sup\;\omega \epsilon R{\left\|H_{j}\left(e-i\omega \right)\right\|}_{2} \end{array}$$

where $\sup_{\omega \epsilon \mathbb{R}}$ denotes the supremum or least upper bound over all real frequencies ω and σ_*max*_ denotes the maximum singular value of the vector ***H***_***j***_. The third equality is due to the fact ***H***_*j*_(*e* − *iω*) that is a column vector.

**Figure 4:**
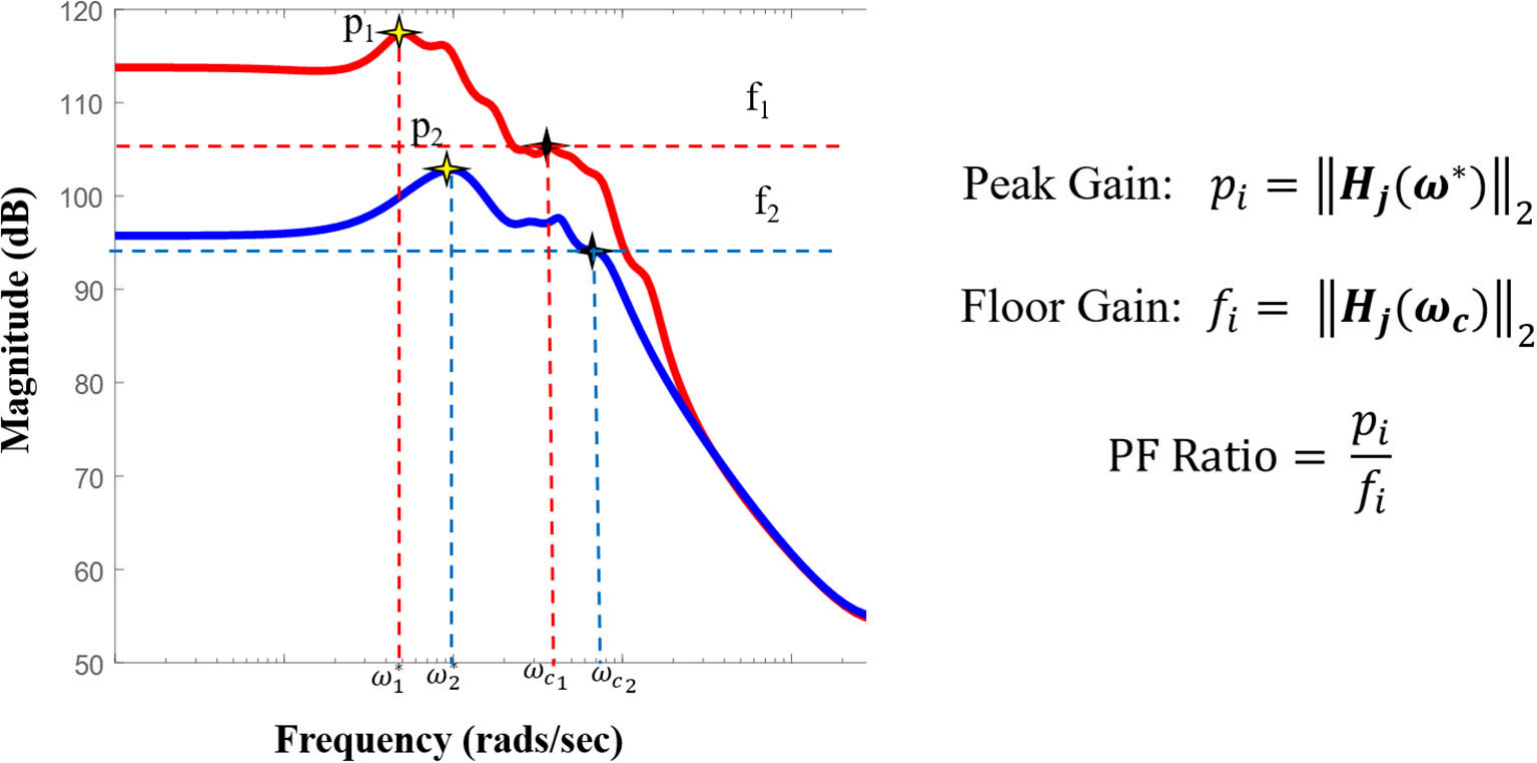
Pictorial representation of the PF ratio and its calculation. Representative bode plot of two transfer function models, where red denotes a clinically annotated SOZ dataset and blue denotes a dataset stimulated that is not part of the epileptogenic region, with their labeled peak and cutoff frequencies, ω* and ωc, respectively. The cutoff frequency is defined as the frequency for which the magnitude is 3dB less than the gain at frequency 0, ω = 0 (DC gain).

For each system gain there is a frequency ω^*^ at which this maximum gain was achieved, ‖ ***H***_*j*_(ω^*^) ‖_∞_. To quantify the “quick” magnitude drop, we calculated the cut-off frequency at which this occurred, ωc. The cutoff frequency in electrical engineering is the boundary where th energy flowing through a system begins to reduce (55). From here, we then calculated the 2-norm of the frequency response evaluated at this cut-off frequency ωc, ‖ ***H***_*j*_(ω*c*) ‖_2_, and finally the PF ratio as follows:

(6) $$PF_{j}=\frac{{\left\|H_{j}\left(\omega *\right)\right\|}_{2}}{{\left\|H_{j}\left(\text{ω}c\right)\right\|}_{2}}$$

where *j* represents each stimulation electrode pair ω^*^ is the peak frequency at which the maximum system gain was attained, and ωc is the cut-off frequency at which the magnitude response begins to drop (Figure 4).

### Correlating PF Ratios to Epileptogenic Regions

Once the PF ratio was computed for each stimulating pair for each subject, we measured the agreement between our PF ratios and the clinical annotations through a confidence statistic (CS). We defined the CS to be the ratio of the mean of the PF ratios of stimulation pairs in the clinically annotated SOZ and early spread (EP) to the mean of the PF ratios of all other stimulation pairs:

(7) $$\begin{array}{l} CS=\;\frac{\frac{1}{m}\sum_{SOZ\&EP}PF_{j}}{\frac{1}{n-m}\sum_{Other}PF_{j}} \end{array}$$

where *m* is the number of stimulation pairs in the SOZ and EP regions, *n* is the total number of stimulation pairs, and *other* is all the stimulation pairs not in the SOZ or EP. We expected the highest GN ratios to closely match the clinical annotated SOZ in patients with a lower clinical complexity score (CCLow). That is we expected higher confidence statistics in patients with lower clinical complexity. On the other hand, patients with a higher clinical complexity score (CCHigh) may show more disagreement between the model results and the clinical notations, resulting in lower and more variable confidence statistics.

### Correlating CCEPs Amplitude to Epileptogenic Regions

In the current SPES literature, there are numerous methods for CCEP analysis. The most common practice is visual inspection of the peak response amplitude, more precisely, the N1 response. The N1 responses are early sharp negative responses occurring anywhere from 10 to 30 ms post stimulation and are believed to reflect the direct structural connections (Figure 2) (11). For our study, to be able to compare the N1 response with our PF ratio, we calculated the N1 peak for all evoked potentials. This was done after the preprocessing of our data in which we looked at a window 10 ms before and 30 ms after the onset of stimulation. Within that time frame, the maximum absolute peak amplitude was calculated, which we called the N1 peak for all output contacts. We then calculated our confidence statistic using the N1 peak as well.

## Results

### Transfer Function Models Reconstruct CCEPs

**Figure 5:**
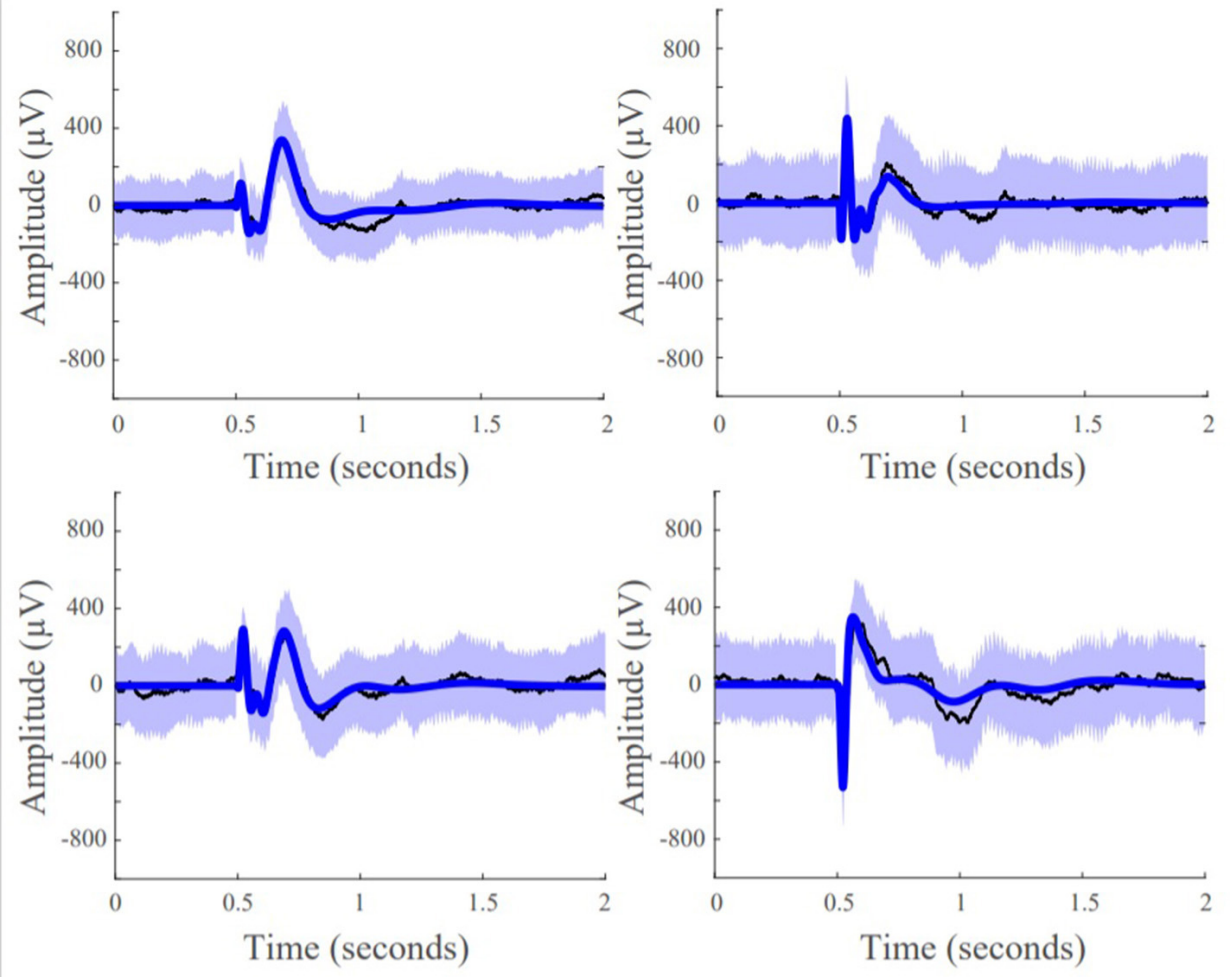
Model fits of four representative responsive channels from patient data showing SIMO transfer function models capture CCEP responses in recording electrodes. Black lines are the average the evoked responses, blue lines are the model reconstruction, and the purple boundaries denotes one standard deviation.

We first assessed whether the SIMO transfer function models were able to accurately reconstruct CCEPs by calculating the percentage of data points that lied within the 95% confidence interval of the mean from the 50 stimulation trials. This resulted in an average concordance of 92.96% indicating that our models were able to accurately reconstruct the mean waveforms of our data, capturing the input-output behavior of CCEPs under SPES (Figure 5).

### Higher PF Ratios in the Seizure Onset Zone for Low Clinical Complexity Cases

In cases of low clinical complexity, our expectation was that our models would agree with the clinical annotations, and in cases of high clinical complexity there would be high variability of agreement between our model statistics and clinical annotations. We further hypothesized that successful surgical outcomes will show high agreement regardless of clinical complexity.

**Figure 6:**
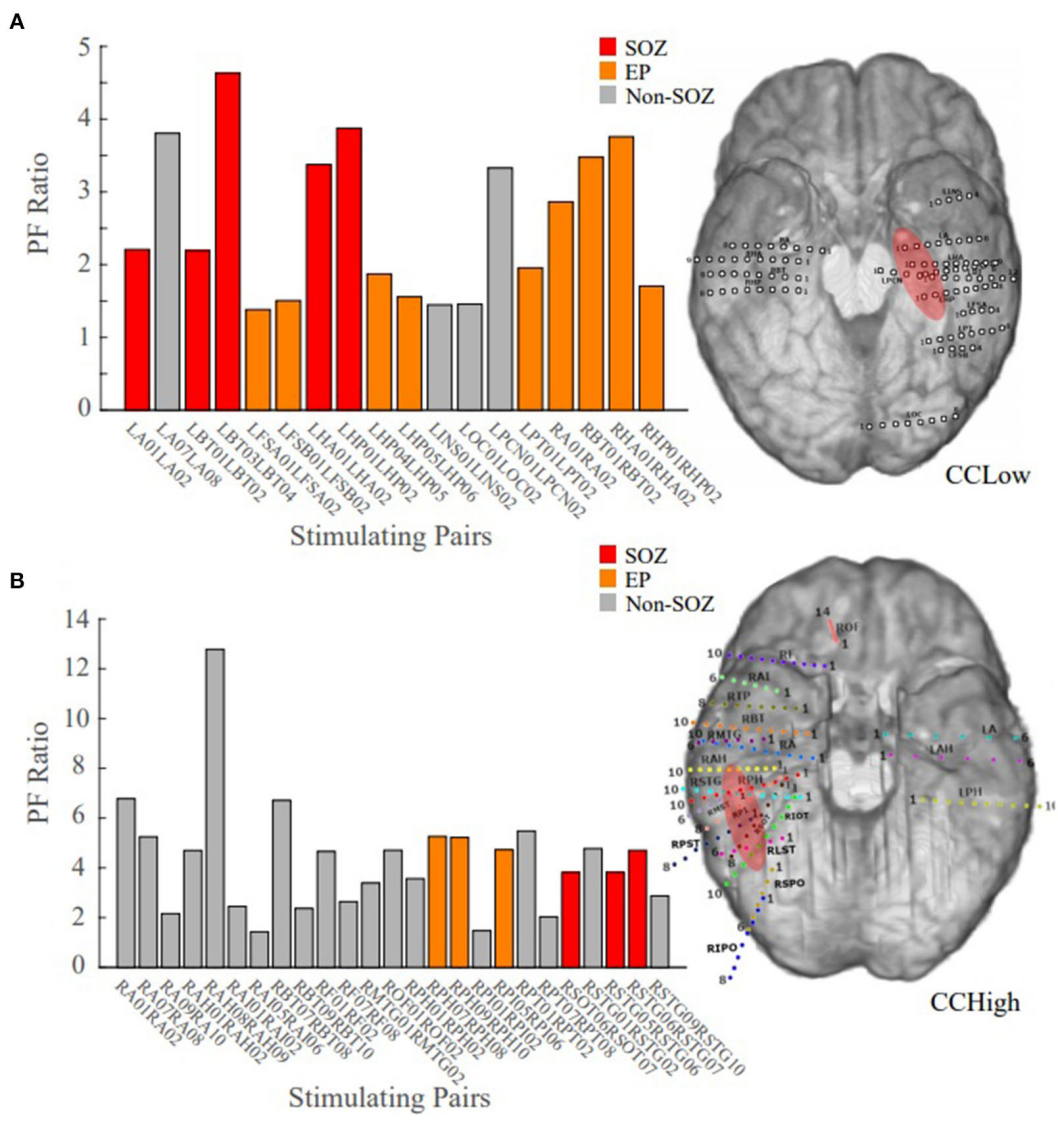
Bar plot of the PF ratios, where red indicates electrode pairs in the clinically annotated seizure onset zone (SOZ), orange indicates early spread (EP), and gray represents all others. To the right of the bar graphs are the electrode implantation maps for each patient with the clinical annotated SOZ denoted in red. **(A)** PF ratios of representative “model success” Patient 13 dataset; high PF ratio values closely correspond with SOZ and EP regions. **(B)** PF ratios of representative “model disagreement” Patient 14A dataset; SOZ and EP PF ratios are indistinguishable from non-epileptogenic regions.

Patient 13 is an example of a low clinical complexity case (CCLow) and Patient 14 is an example of a high clinical complexity case (CCHigh) (Figure 6). These two cases show alignment with our hypothesis in the low clinical complexity cases, our PF ratios are the highest in the areas of the clinical annotated SOZ as well as early spread regions resulting in a higher confidence statistic, while in the higher complexity case, the highest PF ratios are not in areas deemed to be part of the epileptogenic network.

In Patient 13, the largest PF ratios were associated with stimulation pairs that were in the SOZ (Figure 6A). Further, most other contact pairs in the EP (orange) also yielded high PF ratios, while the two electrodes pairs believed to not be part of the epileptic network (gray) had the smallest PF ratios. The high degree of agreement between our model gains and the clinical annotations resulted in a CS of 1.034.

In Patient 14, the largest PF ratios were in areas outside of the clinical annotations (Figure 6B), where the average PF ratio of 27.550 for the SOZ and EP electrodes and an average GN ratio of 80.2357 in all other electrode pairs.

### Faster Magnitude Roll-Offs in Successful Patient Outcomes

**Figure 7:**
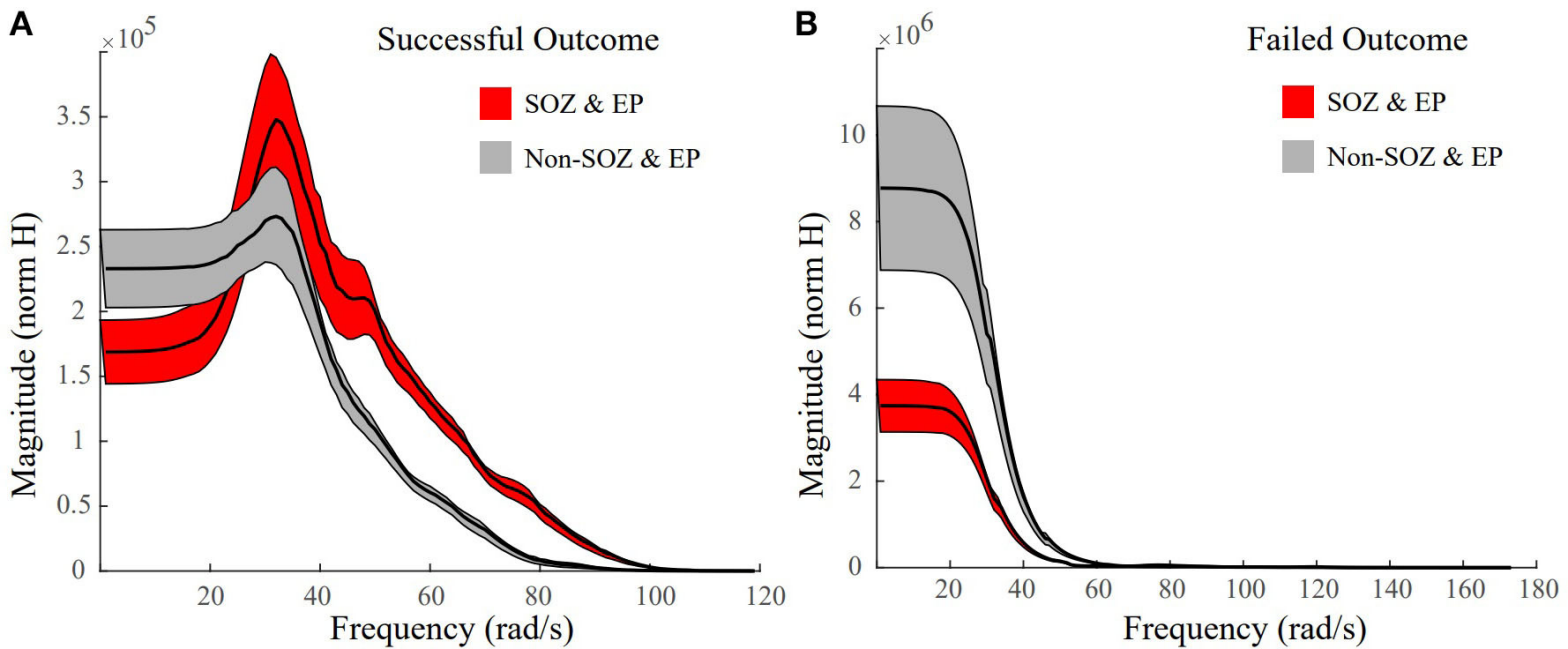
Representative frequency response plots of a successful and failed surgical outcome where red denotes SOZ and EP stimulated datasets, grey denotes non-SOZ and EP stimulated datasets, black is the mean frequency response, and the shaded regions denote ±2 standard error. **(A)** Frequency response plot of the SOZ & EP stimulated datasets vs the non-SOZ & EP stimulated datasets for successful surgical outcome Patient 18. The SOZ & EP stimulated datasets show a larger peak gain and a bigger roll off than the non-SOZ & EP counterparts. **(B)** Frequency response plot of the SOZ & EP stimulated datasets vs the non-SOZ & EP stimulated datasets for failed surgical outcome Patient 2.

In successful surgical outcomes, where clinicians were able to accurately localize the SOZ, we anticipated frequency response plots similar to the ones in Figure 4, where the SOZ stimulated dataset would have a high peak gain and a roll-off in magnitude compared to the non-SOZ stimulated datasets. The mean frequency response plot of the SOZ and EP stimulated datasets of Patient 18, a surgical success, had a high peak gain and a big roll-off compared to the non-EZ stimulated datasets (Figure 7A). In the failed surgical outcome case of Patient 2 (Figure 7B), the SOZ stimulated datasets not only had a very small peak gain, but a rather slow roll-off as well. However, the non-SOZ stimulated datasets had overall the highest peak gains and incredibly fast roll-offs, suggesting that these datasets may be part of the epileptogenic region.

### Correlating Surgical Outcomes to PF Ratios

**Figure 8:**
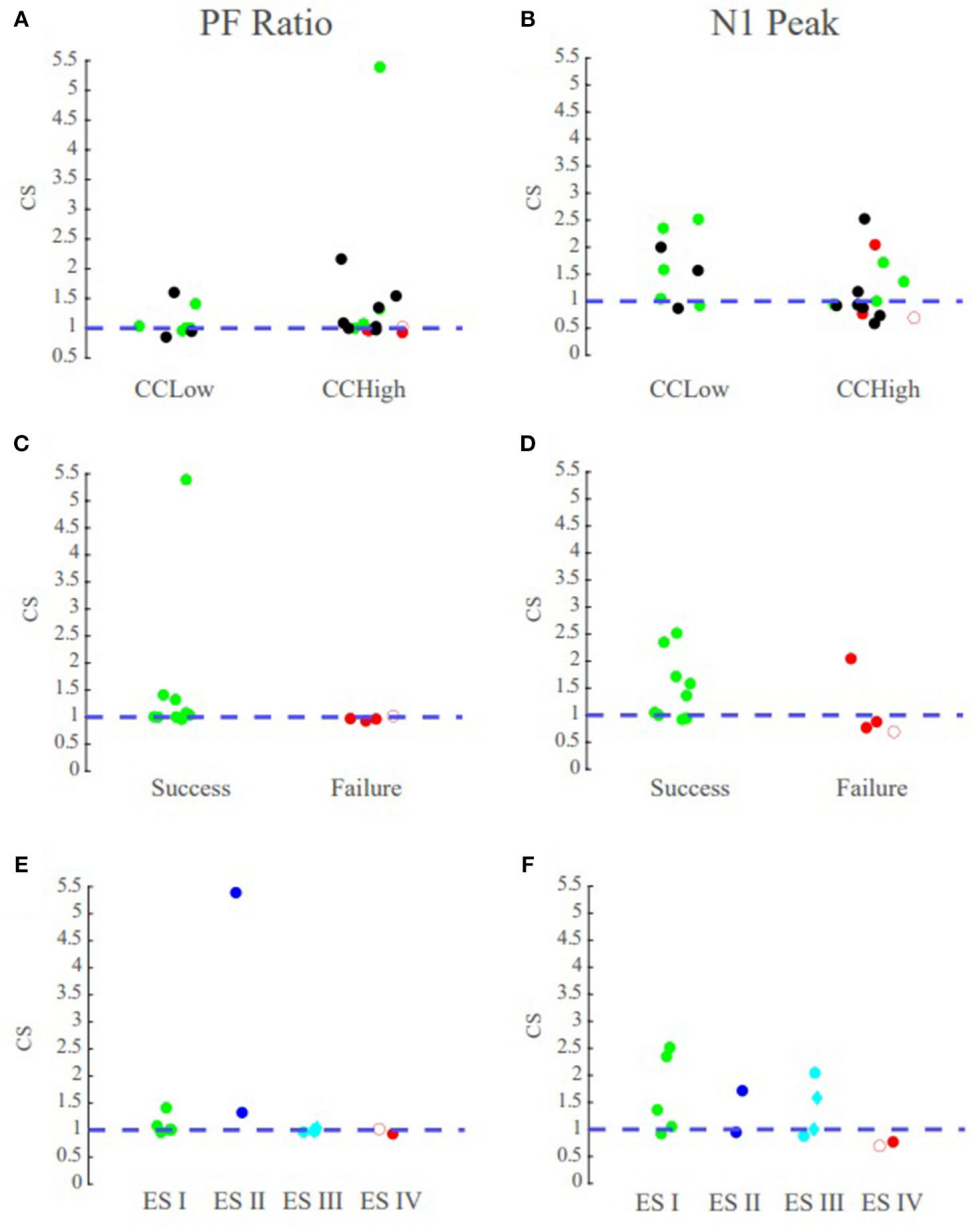
PF ratio confidence statistic reflects surgical outcome. Green circles denote those patients with successful surgical outcomes, red denotes those with failed surgical outcomes, and black denotes datasets that currently have no surgical outcome. The blue dotted line denotes the boundary for the degree of agreement between clinical annotations and the performance metrics. Diamond shape indicates RNS patients and open circle indicates outlier Patient 5. Top row contains the confidence statistic plots for all datasets. Middle row are the confidence statistic plots for surgical patients categorized by surgical success and failures. Bottom row has the confidence statistic plots for surgical patients categorized by Engel Score. **(A)** The confidence statistic plot for the PF ratio for all datasets. CCLow patients often have CS > 1, and surgical success nearly always have CS values >1. **(B)** The confidence statistic plot for N1 peak for all datasets. Less distinction is provided between groups according to the N1 amplitude as compared to system gain. **(C,D)** Confidence statistic plots for surgical patients categorized by surgical success/failure for **(C)** PF ratio **(D)** and N1 peak. **(E,F)** Confidence statistic plot for surgical patients categorized by Engel score for **(E)** PF ratio and **(F)** N1 peak.

We have summarized our findings for all 22 patients with three different scatter plots (Figure 8). The first plot displays the confidence statistic for all patients classified in terms of their clinical complexity, confidence statistic, and surgical outcome if available (Figure 8A). The second plot displays the confidence statistic for only those patients with surgical outcome classified in terms of their confidence statistic and either surgical failure or success (Figure 8B). Finally, the last scatter plot again displays the confidence statistic for patients with surgical outcome but has now separated outcomes in terms of the Engel Score (Figure 8C). The dotted line indicates the degree of agreement boundary, where CS values above the line indicate patients whose highest PF ratios agreed most with the clinically annotated SOZ and EP regions, and thereby implying a greater chance of surgical success, while those CS values below the line indicate patients whose highest PF ratios varied most with clinical annotations, and potentially imply a greater chance of surgical failure.

We tested whether the transfer function models were able to not only localize the SOZ, but also anticipate the surgical outcome for seizure freedom. Overall, in the MRE patients who underwent resective surgery and had a successful outcome (ES I and ES II), indicating the clinicians were able to successfully localize the SOZ, our models had the highest PF ratios in the clinically annotated SOZ and therefore a high confidence statistic (CS ≥ 1), irrespective of the clinical complexity (Figure 8C). In the surgical resection cases that resulted in poor seizure outcomes (ES III and ES IV), suggesting the clinicians were unable to precisely and accurately localize the SOZ, our models exhibited low PF ratios in the clinically annotated SOZ and larger PF ratio values in areas that were not part of the clinically annotated SOZ (CS < 1) (Figure 8C). Thus, we conjecture that in patients that have undergone surgical resection/ablation, a high concordance between our models and clinical annotations would suggest seizure freedom, while large variations between our models and annotations would suggest a poor surgical outcome.

### PF Ratios vs. N1 Peaks

To determine the efficacy of our system metric over the current CCEP analysis through visual inspection of the N1 amplitude, we analyzed the correlation between PF ratio and peak amplitude as well as the confidence statistic for N1. The confidence statistic for N1 demonstrated slightly poorer performance in the classification of surgical outcomes than the PF ratio (Figure 8B). In the CCLow cases, one of the datasets (Patient 4) which has a successful surgical outcome has a CS < 1, while in the CCHigh case Patient 2, which had an unsuccessful surgical result, has a CS > 1 (Figure 8B). Additionally, the Pearson correlation between PF ratios and N1 peak amplitude for all datasets averaged 0.1515 ± 0.1676, indicating little correspondence between the metrics (Supplementary Figure 1).

## Discussion

Patient specific dynamical network models were built from SPES data and analyzed for a population of medically refractory epilepsy patients that were admitted to the Johns Hopkins Hospital. These patients were admitted for the localization of their seizure network for the possibility of seizure freedom via surgical resection. As epileptic seizures are believed to result from a pathologically connected brain network with epileptic foci (56), we conjectured that the analysis of intracranial EEG data in response to stimulation in the context of dynamic networks would provide an advantage to current localization techniques that are based on passive iEEG. SPES provides an opportunity to actively perturb the brain, and then capture and analyze the rich dynamics of the iEEG network to localize the SOZ.

### Dynamical Network Models for the Localization of the SOZ

Work done by A. Li et al. (54) showed that an epileptic brain can be modeled as a network that is on the verge of instability, where a small perturbation can result in the manifestation of a seizure. There are nodes within this network that are potentially more “fragile” than others, corresponding to the brain regions associated with the onset of the seizure. The fragility of these network nodes makes them susceptible to small perturbations, evoking a significant response or disturbance in the network, possibly initiating a seizure. We hypothesized that these “fragile” nodes should produce large responses, responses larger than the other nodes within the network. It is also known that seizure spread is specified by impaired excitation and inhibition balance, suggesting that large responses may be a potential biomarker of this imbalance (57–59).

We used transfer function models to analyze the responses of the network to SPES. One performance metric of these models that can quantify and characterize this notion is the peak gain of the system and the cutoff frequency at which the magnitude drops by half. This can be calculated through a ratio of the $H_{\infty }$ norm and the 2-norm of the associated frequency response evaluated at the cutoff frequency. Our conjecture was that those electrode pairs with the highest PF ratio of peak gain to cutoff gain to input norm would correspond to the electrode pairs in the clinically annotated SOZ, particularly for patients with low clinical complexity and successful surgical outcomes.

### PF Ratios Correlate to Clinical Annotations

We hypothesized that the areas involved in the epileptogenic region, such as SOZ and EP, when stimulated, would produce the largest system gains and the biggest response drop-offs as compared to areas not involved in the epileptogenic network. We further conjectured that for those patients whose epilepsy was due to a lesion, had a focal onset, or originated solely in the temporal region (CCLow), the clinicians would be able to identify the SOZ accurately and completely. This would suggest that, in our models, those electrode pairs in the clinically annotated SOZ and EP, should have large gain values *and* small floor gain values, when compared to other electrode pairs. Therefore, we expected to see a higher degree of agreement between our PF ratios and the clinical annotations, resulting in a confidence statistic ≥1. However, for those patients whose epilepsy was non-lesional, multifocal, and extratemporal, (CCHigh), we speculated that the clinicians had a more difficult time precisely locating the SOZ and early spread regions. Thus, we expected in these cases for our PF ratios to have more variations and potential disagreements with the clinical annotations (more variable CS), possibly highlighting areas that may have been overlooked or that could not be captured with the current localization methods. Our models may also be able to predict which patients will have surgical success and which will fail, depending on the level of disagreement between the model and the clinical annotations. A larger discordance would indicate a more complex case and the increased likelihood of a failed outcome.

We explored the relationship between the ratio of the system gains and the input norms of our transfer function models to regions of epileptogenic interest. Overall, we found that the patient cases classified of lower clinical complexity tended to have the highest PF ratio in the electrodes clinically marked as SOZ. If not in the SOZ, often electrode pairs with higher PF ratios belonged to locations that were of interest, such as the EP. For example, Patient 13 had been classified as CCLow because the patient presented with a focal encephalomalacia of the inferior temporal lobe. This lesion, in conjunction with the patient's seizure semiology and iEEG recordings made the localization of the SOZ and early spread regions more straightforward for the clinicians.

On the other hand, as the clinical complexity increased, the discrepancies between the model PF ratios and the clinically annotated SOZs also increased. In Patient 14, the electrode pairs in the clinically annotated SOZ and EP, yielded some of the smallest PF ratio values (Figure 6B). However, this patient has been admitted to the JHH EMU on two separate occasions for localization of seizure onset. During both stays, the clinicians were unable to localize the SOZ, requiring a third visit with the implantation of a grid. The inability to localize this patient's seizures implies that though there is disagreement between the clinical annotations and the PF ratios, our model may be identifying regions of interest that the clinicians were unable to identify through individual iEEG channel inspection.

### Large Magnitude Drop Offs Correlate to Epileptogenic Regions

Studying the mean frequency responses of our systems, we explored properties that may indicate the epileptogenic zone. We observed that the frequency responses of the SOZ stimulated datasets in successful surgical outcomes, had not only some of the largest system gains, but also some of the quickest and biggest magnitude drops, especially when compared to their non-SOZ counterparts (Figure 7A). This difference became even more striking when comparing the mean SOZ stimulated datasets vs the mean non-SOZ stimulated datasets for a failed surgical outcome case. In this instance, the clinically annotated SOZ stimulated datasets had some of the smallest peak gains and some of the slower, smaller magnitude drop offs, while the non-SOZ stimulated datasets had very high peak gains and steep drop offs (Figure 7B). This may suggest a resonance-like property of cortical networks that can generate seizures if triggered by a periodic stimulation at a particular frequency. This is certainly the case for photosensitive epilepsy (60), where flashing stimuli (in time or space) at a particular frequency may cause a seizure. Our findings suggest that resonance may be more prevalent in all types of epilepsy.

### PF Ratios Reflect Surgical Outcomes

**Figure 9:**
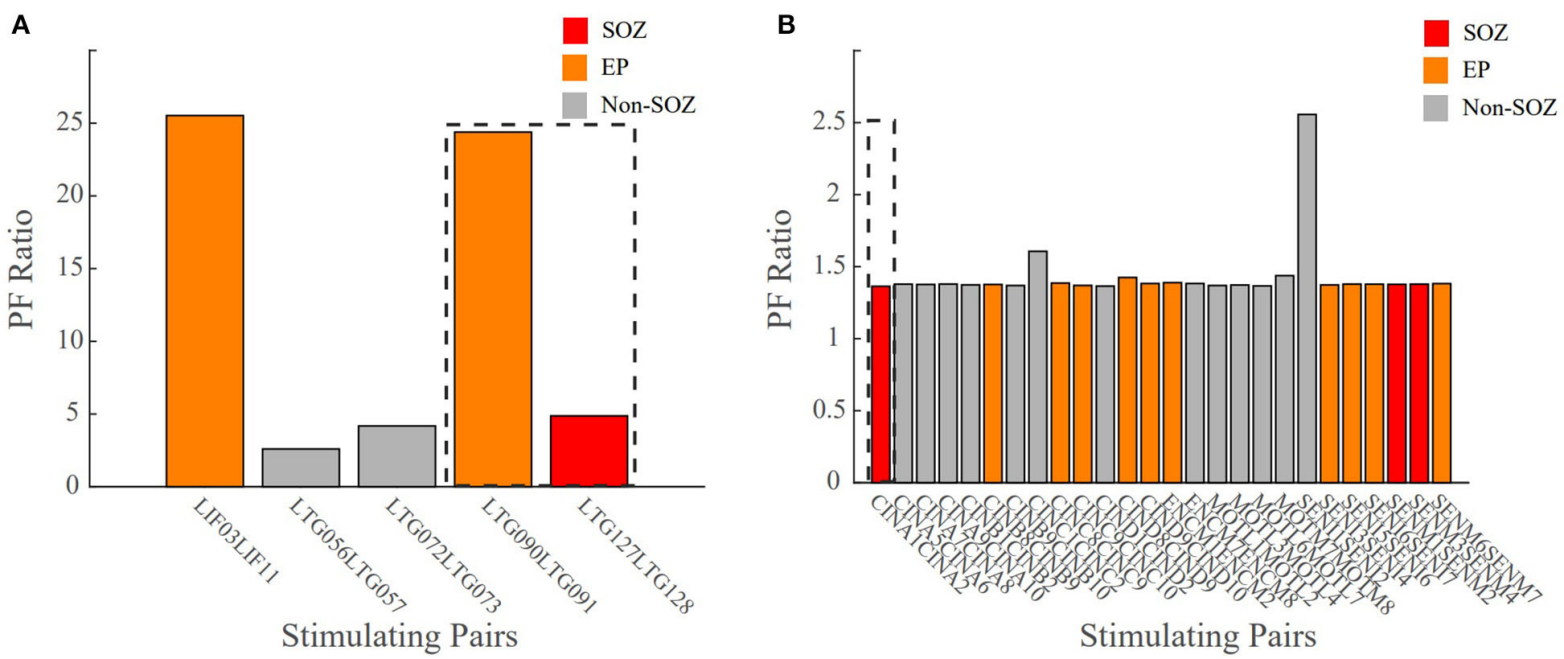
Bar plot of the PF ratio transfer functions for two patient datasets with surgical outcome data. Dashed lines indicate the areas of resection. **(A)** CCLow Patient 3 who now is seizure free (ESII), **(B)** CCHigh Patient 7 who still has seizures (ES IV). Red indicates electrode pairs in the clinically annotated seizure onset zone (SOZ), orange is early spread (EP), and gray are all others. In surgical success, the resected regions closely matched the clinically annotated SOZ regions with high PF ratio values.

A true test for SOZ localization algorithms is in their ability to predict surgical outcomes. We defined a successful surgical outcome to be those patients with an Engel score or I and II, or if a responsive neurostimulation (RNS) device was implanted, an Engel score of III was considered a success. A failed surgical outcome was defined as those patients who had surgical resection or ablation and received an Engel score of III or IV. Patient 3 was categorized as CCLow and now has seizure freedom (ES II) (Figure 9A). The clinicians identified electrode pairs LIF03/11, LTG09/91 and LTG127128 as those they believed to be the in the EP (orange) and SOZ (red), respectively. Our model revealed that LTG90/91 and LIF03/11 had the highest PF ratios out of all electrode pairs. The resected areas included the SOZ contact LTG127/128 and EP contact LTG90/91, which had one of the highest PF ratios. Which had the two highest PF ratios. Given the surgical outcome of ES II, this demonstrates the agreement between our model and clinical annotations in patient cases of low clinical complexity and the correlation to successful surgical outcomes.

In unsuccessful surgical outcomes (ES III and ES IV), we hypothesized the highest PF ratios would be in regions not labeled as the SOZ. Patient 7 was a difficult case (CCHigh) who, despite a laser ablation at contact CINA1-2, had no improvement in their seizure frequency (ES IV) (Figure 9B). The low PF ratio in CINA1-2 indicates that our algorithm identified this region of the brain as non-epileptogenic. Moreover, our algorithm identified SENI1-2 as a region that may possibly show epileptogenicity due to the high PF ratio values in that region.

**Figure 10:**
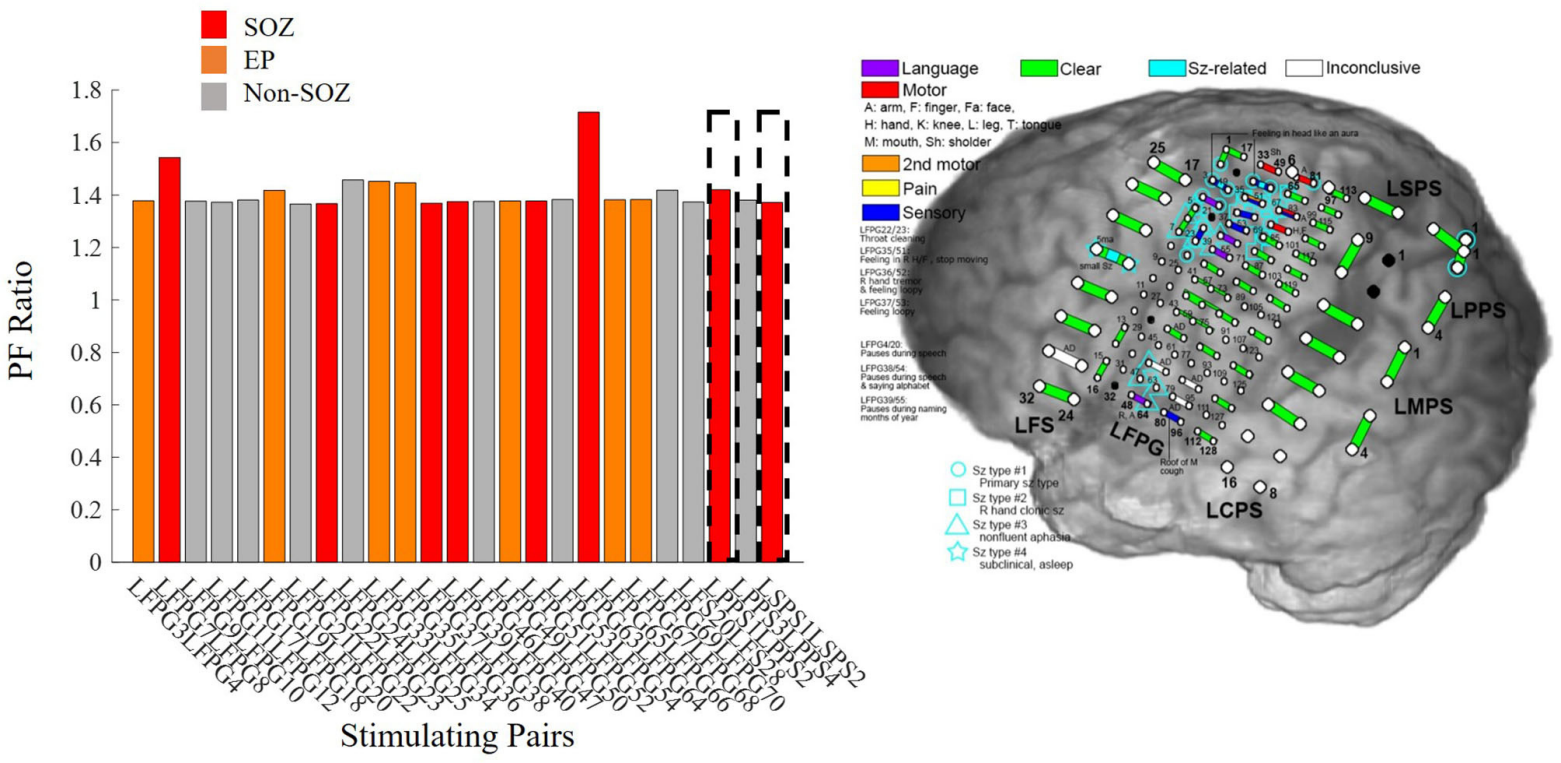
Bar plot of PF ratios for Patient 5 with a high confidence statistic value according to the model, but an unsuccessful surgical outcome. The dotted boxes represent the regions that were resected. Red indicates electrode pairs in the clinically annotated seizure onset zone (SOZ), orange is early spread (EP), and gray are all others. Regions with high PF ratios were not resected because they were in eloquent cortex, possibly explaining the seizure persistence.

There were instances where our model agreed with the clinical annotations resulting in a high confidence statistic, however, the surgical outcome did not result in seizure freedom. Outlier Patient 5 is a patient whose clinically annotated SOZ and EP contacts resulted in high PF ratios which would normally suggest a success (Figure 10). The regions that were resected were those in the SOZ pairs LPPS1/2 and LSPS1/2, which resulted in an unsuccessful surgical outcome (ES IV). However, despite the poor surgical results, the confidence statistic for this patient (Patient 5) was high (CS = 1.0263). Due to part of the SOZ (electrode pair LFP63/64) being located in a language area of the cortex, it was not surgically removed to prevent a functional deficit. The next highest PF ratios not resected were in the EP pairs LFPG33/34 and LFPG35/36, which were also located in the eloquent cortex (motor), making the tissue not viable for resection. Failing to remove the entire SOZ likely caused the failed outcome. This is just one of several examples demonstrating the complexity of diagnosing and treating these patients and more importantly the difficulty of evaluating computational algorithms.

### Study Limitations

The major limitation of this study is the low number of study subjects, particularly those with surgical outcomes. Extending this study to more patients with varying pathologies and epilepsy etiologies, particularly those with surgical outcomes, would increase the power of this study. The inclusion of more surgical outcome data would help to prove the efficacy of the PF ratio and its advantages over the N1 peak. There are also other properties of the transfer function models that need to be explored, such as the phase delay, and pole-zero locations. Potentially, analysis and inclusion of these additional metrics may help more accurately and fully characterize the epileptic network and show the advantage of using the PF ratio for localization of the SOZ, particularly in cases of high clinical complexity.

## Data Availability Statement

The datasets presented in this article are not readily available because, we leave it to the clinical team to decide if the datasets are available upon request. Requests to access the datasets should be directed to Dr. Joon-Yi Kang, jkang50@jhmi.edu.

## Ethics Statement

The studies involving human participants were reviewed and approved by Studies of Patients with Implanted Intracranial Electrodes (IRB 00044461). The patients/participants provided their written informed consent to participate in this study.

## Author Contributions

GK, RS, SS, NC, and JK designed the study. NC, JK, MH, and CC conducted the experiments and collected the data. GK and RS implemented the algorithms, preprocessed the data, performed the data analysis, and wrote the manuscript. SS and JK provided the research ideas and revised the manuscript. All authors revised and approved the final version of the manuscript.

## Conflict of Interest

The authors declare that the research was conducted in the absence of any commercial or financial relationships that could be construed as a potential conflict of interest.
