# Supplementary material for: Transfer Function Models for the Localization of Seizure Onset Zone From Cortico-Cortical Evoked Potentials
Source: Front Neurol. 2020 Dec 10;11:579961. doi: 10.3389/fneur.2020.579961 (PMC7758451; doi:10.3389/fneur.2020.579961)
Supplement: Supplementary file 1 [file Data_Sheet_1.docx]

Supplementary Material


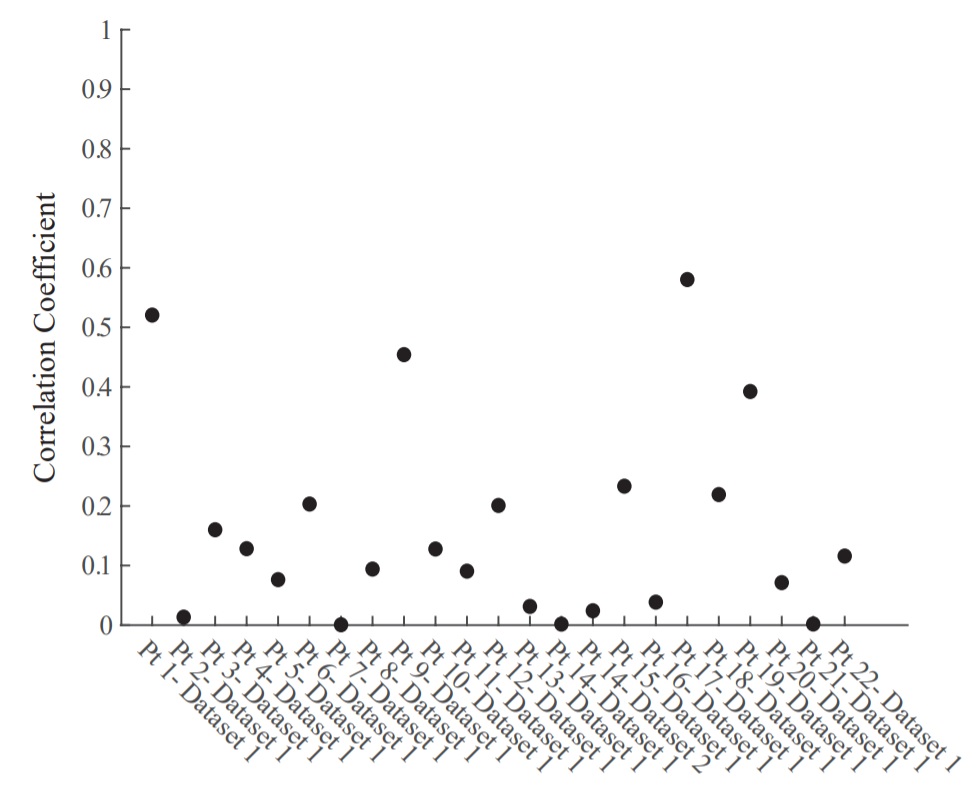


Supplementary Figure 1: Pearson correlation between PF ratio and N1 peak for each dataset.


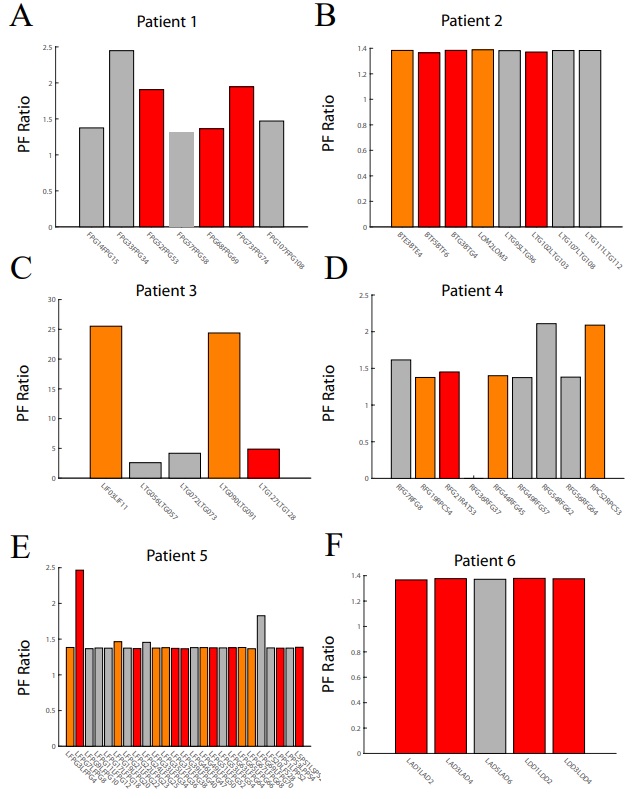


Supplementary Figure 2: PF ratio bar plots for patients 1-6. (**A,B**) Top row are the bar graphs of the PF ratio for Patients 1 & 2, (**C,D**) middle row are patients 3 & 4, and (**E,F**) bottom row are patients 5 & 6. Red indicates electrode pairs in the clinically annotated seizure onset zone (SOZ), orange is early spread (EP), and grey are all others.


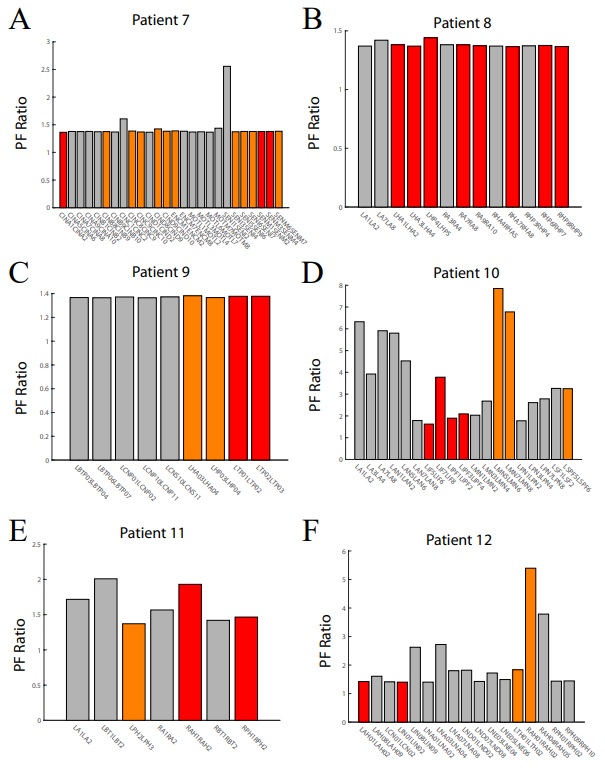


Supplementary Figure 3: PF ratio bar plots for patients 7-12. (**A,B**) Top row are the bar graphs of the PF ratio for Patients 7 & 8, (**C,D**) middle row are patients 9 & 10, and (**E,F**) bottom row are patients 11 & 12. Red indicates electrode pairs in the clinically annotated seizure onset zone (SOZ), orange is early spread (EP), and grey are all others.


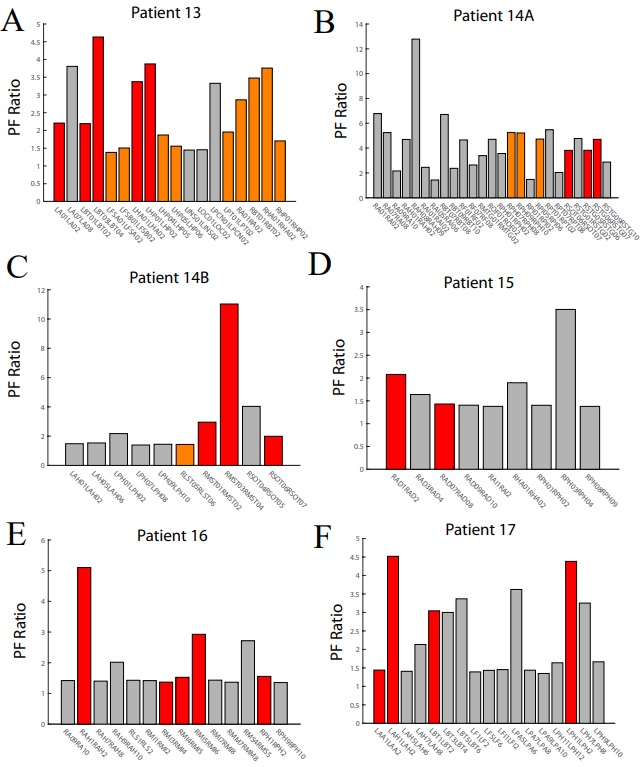


Supplementary Figure 4: PF ratio bar plots for patients 13-17. (**A,B**) Top row are the bar graphs of the PF ratio for Patients 13&14A, (**C,D**) middle row are patients 14B & 15, and (**E,F**) bottom row are patients 16 & 17. Red indicates electrode pairs in the clinically annotated seizure onset zone (SOZ), orange is early spread (EP), and grey are all others.


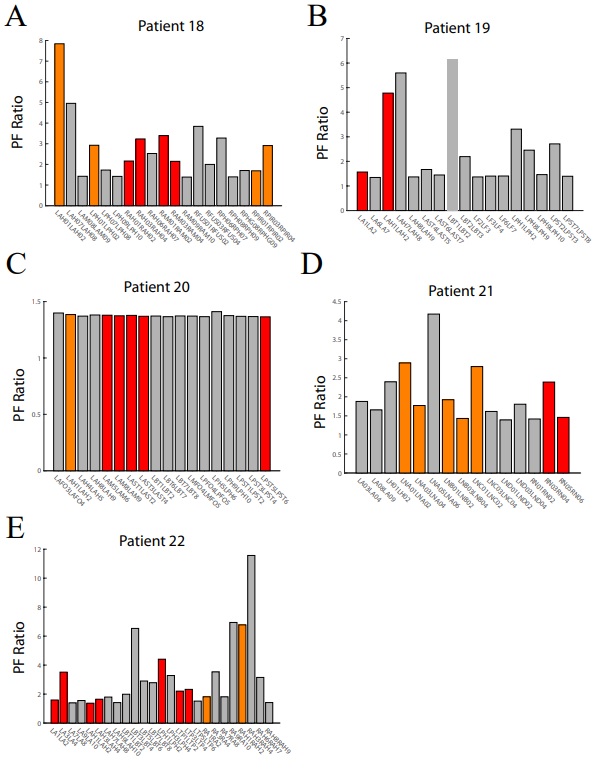


Supplementary Figure 5: PF ratio bar plots for patients 18-22. (**A,B**) Top row are the bar graphs of the PF ratio for Patients 18 & 19, (**C,D**) middle row are patients 20 & 21, and (**E,F**) bottom row is patient 22. Red indicates electrode pairs in the clinically annotated seizure onset zone (SOZ), orange is early spread (EP), yellow is irritative zone (IZ), and grey are all others.
